# Investigating inter-chromosomal regulatory relationships through a comprehensive meta-analysis of matched copy number and transcriptomics data sets

**DOI:** 10.1186/s12864-015-2100-5

**Published:** 2015-11-18

**Authors:** Richard Newton, Lorenz Wernisch

**Affiliations:** Biostatistics Unit, Medical Research Council, Robinson Way, Cambridge, CB2 0SR UK

**Keywords:** Gene regulatory relationship, aCGH, Transcriptomics, Cancer, Meta-analysis, Activation, Repression, Cocitations

## Abstract

**Background:**

Gene regulatory relationships can be inferred using matched array comparative genomics and transcriptomics data sets from cancer samples. The way in which copy numbers of genes in cancer samples are often greatly disrupted works like a natural gene amplification/deletion experiment. There are now a large number of such data sets publicly available making a meta-analysis of the data possible.

**Results:**

We infer inter-chromosomal acting gene regulatory relationships from a meta-analysis of 31 publicly available matched array comparative genomics and transcriptomics data sets in humans. We obtained statistically significant predictions of target genes for 1430 potential regulatory genes. The regulatory relationships being inferred are either direct relationships, of a transcription factor on its target, or indirect ones, through pathways containing intermediate steps. We analyse the predictions in terms of cocitations, both publications which cite a regulator with any of its inferred targets and cocitations of any genes in a target list.

**Conclusions:**

The most striking observation from the results is the greater number of inter-chromosomal regulatory relationships involving repression compared to those involving activation. The complete results of the meta-analysis are presented in the database METAMATCHED. We anticipate that the predictions contained in the database will be useful in informing experiments and in helping to construct networks of regulatory relationships.

**Electronic supplementary material:**

The online version of this article (doi:10.1186/s12864-015-2100-5) contains supplementary material, which is available to authorized users.

## Background

We performed a joint analysis of 31 matched array comparative genomics (aCGH) and transcriptomics human cancer data sets; that is, experiments in which aCGH and transcriptomics arrays have been run on the same samples. We obtained inter-chromosomal acting regulatory relationships inferred from these data sets. By regulatory relationship we mean either a direct relationship, of a transcription factor on its target gene, or a very indirect one, through a pathway containing many intermediate regulatory steps.

For tumour samples, aCGH microarrays compare gene copy numbers in the DNA extracted from the cells under investigation to the gene copy numbers in normal control cells, in order to detect gene deletions or gene amplifications (double or more copies of a gene compared to normal). Typically, the DNA is extracted from a tumour sample containing many cells, which may exhibit different alterations in copy number. So for each gene the measured change in copy number is an average for all the cells in the sample and will, in general, be fractional rather than integer. The gene expression experiments also utilise microarrays, but measure the abundance of mRNA. Reviews of matched experiments, their analysis and uses can be found in Huang et al. [1] and Lahti et al. [2].

The way in which copy numbers of genes in cancer samples are often greatly disrupted works like a natural gene amplification/deletion experiment, so when transcriptomics data is also available for the same samples, then information can be gleaned on how changes in a gene’s copy number affects that gene’s expression. In addition, but more challenging, the data can be used to investigate whether the change in a gene’s copy number, and concomitant change in expression, affects the expression of other genes, hence inferring regulatory relationships.

Predicting regulatory relationships from this type of data faces multiple obstacles. The signal is quite weak, due to the noise in the data, and the complexity of the biology involved presents a number of additional problems. There can be a high degree of non-linearity in the relationship between copy number and expression, in fact gene amplification in some circumstances may actually reduce the expression of that gene [1, 3, 4]. One cause of non-linearity in regulatory relationships is that amplification or deletion of genes can result in alternative regulatory pathways being activated. A further problem for the analysis is that the results for a potential regulator can be confounded by coamplified/codeleted genes situated in the same region of the genome, and it is for this reason that we concentrate on inter-chromosomal acting regulatory relationships.

Many genes that have altered copy number in one cancer type are found to have altered copy number in other cancer types [5], so combining data sets from multiple cancer types should help reinforce any information within the data on regulator-target relationships. The 31 data sets used for the inference are comprised of several types of human cancer and a total of 2574 samples. Heterogeneity between cancer types does mitigate any improvement, however we have shown experimentally in Goh et al. [6] and computationally in Newton & Wernisch [7] that there is useful signal on regulatory relationships within the data. Inferences from a meta-analysis of matched data sets were presented in Newton & Wernisch [7] for a few potential regulators whereas the present analysis makes available the results for all potential regulators in the matched data sets. The complete results of the meta-analysis are presented in the METAMATCHED database (http://sysbio.mrc-bsu.cam.ac.uk/METAMATCHED).

## Methods

### Data

Table 1 lists the 29 experiments used in the meta-analysis. If an experiment used two different expression platforms then the samples for each expression platform are treated as a separate data set. This is done in order to avoid the possibility of spurious correlations which may be caused by systematic distortions or shifts between the two sets of expression data. This situation pertains to two of the experiments, so these two experiments contribute four data sets to the study, resulting in a total of 31 data sets from the 29 experiments.

**Table 1:** Details of the 31 data sets used in the meta-analysis

Code	GEO	Publication	*N*	*P*	Pathology
parr	GSE20486	Parris et al. 2010 [24]	97	18616	Breast cancer (Diploid)
crow	GSE15134	Crowder et al. 2009 [25]	31	16153	Breast cancer (ER+)
sirc	GSE17907	Sircoulomb et al. 2010 [26]	51	14689	Breast cancer (ERBB2 amplified)
myll	^*a*^	Myllykangas et al. 2008 [27]	46	17050	Gastric cancer
junn	^*a*^	Junnila et al. 2010 [28]	10	16844	Gastric cancer
ch.w	^*b*^	Chitale et al. 2009 [29]	91	10285	Lung adenocarcinoma
ch.s	^*b*^	Chitale et al. 2009 [29]	94	10285	Lung adenocarcinoma
hoac	GSE20154	Goh et al. 2011 [30]	54	14388	Oesophageal adenocarcinoma
zho	GSE29023	Zhou et al. 2012 [31]	115	13697	Multiple myeloma
shai	GSE26089	Shain et al. 2012 [32]	68	14201	Pancreatic cancer
vain	GSE28403	Vainio et al. 2012 [33]	13	10107	Prostate cancer
bott	GSE29211	Bott et al. 2011 [34]	53	10321	Pleural mesothelioma
bekh	GSE23720	Bekhouche et al. 2011 [35]	173	13682	Breast cancer (Inflammatory)
chap	GSE26863	Chapman et al. 2011 [36]	245	13667	Multiple myeloma
ooi	GSE22785	Ooi et al. 2012 [37]	14	10091	Neuroblastoma
brag	GSE12668	Braggio et al. 2009 [38]	11	10310	Waldenström’s macroglobulinemia
jons	GSE22133	Jönsson et al. 2010 [39]	356	4183	Breast cancer
mura	GSE24707	Muranen et al. 2011 [40]	47	4472	Breast cancer
lin1	GSE19915	Lindgren et al. 2010 [41]	72	4965	Urothelial carcinoma
beck	GSE17555	Beck et al. 2010 [42]	18	12174	Leiomyosarcoma
toed	GSE18166	Toedt et al. 2011 [43]	74	4289	Astrocytic gliomas
ell	GSE35191	Ellis et al. 2012 [44]	124	13569	Breast cancer
gra.1	GSE35988	Grasso et al. 2012 [45]	85	12849	Prostate cancer
gra.2	GSE35988	Grasso et al. 2012 [45]	34	12813	Prostate cancer
lenz	GSE11318	Lenz et al. 2009 [46]	203	15212	Lymphoma
lin2	GSE32549	Lindgren et al. 2012 [47]	131	8450	Urothelial carcinoma
micc	GSE38230	Micci et al. 2013 [48]	12	16657	Vulva squamous cell carcinoma
tayl	GSE21032	Taylor et al. 2010 [49]	155	14572	Prostate cancer
coco	GSE25711 ^*c*^	Coco et al. 2012 [50]	36	4394	Neuroblastoma
med	GSE14079	Medina et al. 2009 [51]	8	6376	Lung cancer
przy	GSE54188	Przybyl et al. 2014 [52]	53	17032	Synovial sarcoma

GEO = Gene Expression Omnibus data set reference (http://www.ncbi.nlm.nih.gov/geo/), *N* = Number of samples, *P* = Number of matched probes, ^*a*^http://www.cangem.org/, ^*b*^ http: //cbio.mskcc.org/Public/lung_array_data/, ^*c*^Expression data in ArrayExpress (http://www.ebi.ac.uk/arrayexpress/): E-TABM-38, E-MTAB-161

The aCGH data was location and scale normalized using the median and mad, as was the expression data. The aCGH and expression probes were mapped by the gene names of probes to give the maximum number of probes with corresponding aCGH and expression profiles. If necessary probe gene names were converted from synonyms to standard gene names using the database of the HUGO Gene Nomenclature Committee (HGNC) [8]. If there was more than one probe for any gene name then the median value of the probes was taken to represent that gene name. Note that the aCGH data was not thresholded so that, in general, fractional rather than integer aCGH values were used in the analysis. Fractional variations in copy number occur because of the heterogeneity of the cancer samples being studied. By using matched aCGH and expression profiles we eliminated the effects of a sample’s heterogeneity considering that both sets of data were affected equally.

### Analysis

#### Introduction

Full details of the algorithm used and a diagram illustrating the steps involved in the analysis can be found in Newton & Wernisch [7] and in Goh et al. [6], where the code, written in the R statistical environment [9], can also be found. Here we provide a summary of the analysis methods used.

We use a relatively straight-forward method based on correlations which provides a robust method for analysing relationships amongst large amounts of data of unknown complexities. More sophisticated network inference methods are generally much more susceptible to noise and heterogeneity between data sets. The great strength of our simple approach is that it avoids the confounding that can occur when expression data alone is used in the analysis.

We define a ‘regulating gene’ as one whose up or down expression change has a direct or indirect effect on the up or down regulation of a ‘target gene’. Primary candidates for regulating genes are genes having significant correlated changes in their mRNA expression levels following copy number alterations. Potential *target* genes of a regulating gene are those genes with significant correlation between the expression changes of the *target* gene and the aCGH profile of the regulating gene.

We first describe the methods adopted for identifying genes worth investigating as potential regulators. We then describe how we identify potential regulator-target relationships for these genes. We use Spearman correlation throughout the analysis.

#### Identifying potential regulators

In order to identify genes worth investigating as potential regulators we focus on genes that have a high correlation between their copy number and their gene expression.

In the first instance, 31 Spearman rank correlations (from the 31 data sets), and their *p*-values for being greater than zero, were calculated for each gene (R function cor.test). These 31 correlation *p*-values were combined for each gene into a single *p*-value statistic using Fisher’s method (R function survcomp::combine.test). In order not to rely on any statistical assumptions we obtained a null distribution of combined *p*-value statistics through permutation of gene identifiers (see below). The resulting *p*-values for each gene were finally corrected for multiple testing by the Benjamini-Hochberg (B-H) method, to give a false discovery rate (fdr) for each gene based on its aCGH/expression correlations in the 31 data sets. In the following the Benjamini-Hochberg adjusted *p*-values are referred to as adjusted *p*-values and are now fdr values rather than *p*-values in the sense of a type I error.

We were also interested in how many, and which, of the 31 data sets indicated an aCGH/expression correlation. This was assessed for each of the genes using an arbitrary threshold of 0.05 on a gene’s 31 correlation *p*-values (after the correlation *p*-values for each data set had been adjusted for multiple testing).

To generate the null distribution, 5·10^6^ permutations of gene identifiers were generated for each data set and the above procedure, using Fisher’s method, for obtaining combined *p*-value statistics repeated. In practice only a minority of genes are present in all 31 data sets. In general a gene will be present in less than 31 data sets, hence we generated 31 null distributions for *n* combined *p*-values, *n* from 1 to 31.

Altogether there were 12,674 genes considered worth investigating as potential regulators (out of the 19,391 genes which occur in at least one of the data sets), having significant correlation (adjusted *p*-value < 0.05) between copy number profile and gene expression in at least one of the data sets.

#### Predicting regulator-target relationships

After we had identified genes worth investigating as potential regulators we looked for potential target genes of these regulators. Expression changes of a potential target gene must correlate highly with its regulating gene’s aCGH profile. Only inter-chromosomal acting relationships were investigated.

The correlation tests were similar to those in the previous section to find potential regulators but with three additions. Firstly, we tested separately the two alternative hypotheses: that the correlation of a regulator-target pair is greater than zero and that the correlation is less than zero, and we generated separate null distributions for the two conditions. Secondly, for each potential regulator only those data sets were included in the analysis for which that regulator had a significant self aCGH/expression correlation. Thirdly, since we were only interested in inter-chromosomal acting relationships the null distributions were derived using potentially inter-chromosomal acting gene pairs.

Just because a gene appears in a regulator’s list of predicted targets, does not mean that regulator is the most probable regulator for that target. Therefore, for each potential regulator, any predicted inter-chromosomal acted targets were removed if the data indicated an alternative, more probable gene as its regulator. This procedure was found to be important, reducing the number of predicted targets in most cases.

There are however two different criteria that could be used to denote a gene as the most probable regulator of a target gene. Most obviously the lowest adjusted *p*-value from the meta-analysis could be used as the criterion. However there are examples where the regulator with the lowest meta-analysis adjusted *p*-value only has a significant correlation with the target in one of the data sets, when there are other potential regulators that do have higher meta-analysis adjusted *p*-values, but have significant correlations with the target in more data sets. In this analysis we provide results for both criteria for selecting the most probable regulator, the lowest adjusted *p*-value from the meta-analysis and the greatest number of data sets with significant correlations.

#### Co-citations

Co-citation analysis was performed using the Bioconductor [10] package org.Hs.eg.db [11] (version 3.1.2) and functions from the package CoCiteStats [12]. org.Hs.eg.db links a gene identifier to Pubmed identifiers [13] for the papers in Pubmed which cite the corresponding gene. The analysis restricts the results to papers which contain fewer than 100 genes, in order to exclude papers that cite very many genes. Whilst the version 3.1.2 of org.Hs.eg.db was used for the work presented in this paper the cocitation content of the database will be updated at each new release of org.Hs.eg.db. The R package igraph [14] was used to generate network graphs showing how cocitations link genes in a target list.

#### Gene ontology annotations

Gene Ontology (GO) [15] annotations for gene lists comprising the regulator and its predicted target were found using the Bioconductor packages GOstats [16] and GO.db [17] (version 3.1.2). Again the GO annotations will be updated for each new release of GO.db.

## Results

### METAMATCHED database

The complete results of the meta-analysis can be found in the METAMATCHED database of inferred regulatory relationships, available at http://sysbio.mrc-bsu.cam.ac.uk/METAMATCHED. The database contains entries for 12,674 potential regulatory genes (those genes having significant correlation between copy number profile and gene expression in at least one of the data sets). The entry for a gene can be found through a search box on the main page. One thousand four hundred thirty of the potential regulators have significant predicted targets (significance level, adjusted *p*-value < 0.1), and these genes are listed as links on the main page. Additional file 1 summarises the results for the 1430 regulators in a spreadsheet. The website can also be used to access information in the database on 19,391 genes *as targets*. When a particular gene is searched for from the main page, links are provided to information on the gene as a regulator (if any) and information on the gene as a target.

A web page for a gene as a regulator gives summary statistics for the gene, namely the number of predicted target genes activated and repressed by the regulator, the number of regulator-target pairs which have cocitations, the total number of papers which cocite at least two genes from a list containing the predicted targets and the regulator, and the statistical significance of finding this number of cocites. The predicted target genes are listed on the web page and there are links to spreadsheets giving more detailed information on each target’s relationship with the regulator, for example, the number of datasets, and in which datasets, the relationship is significant. The papers which cite both the regulator and a target are listed on the web page with their Pubmed links. The papers which cocite genes in the target list are given in a spreadsheet linked to from the web page. If some of the target list are cocited then a network graph will be displayed showing how cocitations link the genes in the target list (the regulator is also included in the graph if it has cocitations with any of the targets). There is also a link to a page with GO annotations of the target list. Further details of the database can be found in Additional file 2.

Consistent coamplification or codeletion of neighbouring potential regulators coupled with the inherent noise in the data can lead to ambiguity in the database as to which of the regulators is actually regulating a particular target gene. So the results files contain columns giving the ‘best’ regulator in the database for each target based on the criterion of minimum *p*-value and the ‘best’ regulator in the database based on the criterion of significance in the most number of data sets. In addition each gene has a web page containing information in the database on the gene *as a target*. This page contains spreadsheets giving all the significant regulators of the target gene in question.

A web page for a gene *as a target* gives the predicted regulator of the target, for both activation and repression, and based on both the criterion of lowest adjusted *p*-value, and on the criterion of significance in the most number of data sets. Adjusted *p*-values are given for each. Primarily due to coamplification/codeletion of genes in the genomic region of the actual regulator there may be a number of significant predicted regulators, and the number of significant predicted regulators is also given on the web page. Details of all the significant predicted regulators for the target can be found in a spreadsheet linked to on the page. The number of the significant predicted regulators cocited in at least one paper with this target is also given and the papers are listed on the web page.

### Statistical synopsis

#### Number of targets

Altogether 1430 potential regulators had at least one predicted target. This is out of 12,674 genes which have significant self aCGH/expression correlation in at least one data set. There are a total of 22,255 predicted regulator-target pairs in the database. The number of predicted targets for a regulator ranged from 1 to 380, the mean value being 16.

**Fig. 1:**
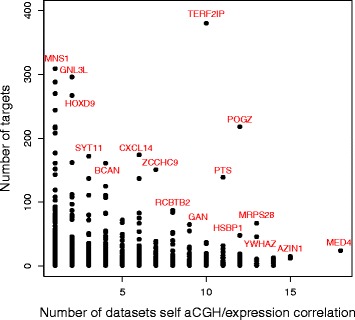
Scatter plot showing, for the 1430 regulators in the database, the number of predicted targets against the number of data sets the regulator shows significant self aCGH/expression correlation. Plot annotated with some of the regulators with large numbers of targets and/or data sets: MED4 - Mediator complex interacts with DNA-binding gene-specific transcription factors to modulate transcription, AZIN1 - Inhibits antizyme-dependent ornithine decarboxylase degradation by binding to antizyme, YWHAZ - Adapter protein implicated in the regulation of a large spectrum of signaling pathways, MRPS28 - Mitochondrial ribosomal protein, POGZ - Zinc finger protein found to interact with the transcription factor SP1, HSBP1 - Overexpression represses the transactivation activity of HSF1, PTS - Biosynthesis of Tetrahydrobiopterin an essential cofactor and regulator of various enzyme activities, TERF2IP - Acts both as a regulator of telomere function and as a transcription regulator, ZBTB43 - Zinc finger and BTB domain containing 43, may be involved in transcriptional regulation, WRB - Receptor, ZCCHC9 - Zinc finger, CCHC domain containing 9, nucleic acid binding, CXCL14 - Belongs to cytokine family which encode proteins involved in immunoregulatory processes, SYT11 - Possibly mediates calcium-dependent regulation of membrane trafficking in synaptic transmission, GNL3L - Essential for ribosomal pre-rRNA processing and cell proliferation, HOXD9 - Belongs to the homeobox family of genes which encode transcription factors, MNS1 - May play a role in the control of meiotic division

Figure 1 shows a scatter plot of the number of predicted targets (activation and repression lists combined) for each regulator against the number of data sets the regulator shows significant self aCGH/expression correlation. The plot is annotated with the names of some of the regulators with large numbers of targets and/or significance in many data sets. The caption gives the function of the genes annotated in the figure, several having a known role in transcription regulation. Of the 1430 regulators, 206 of these are known to be involved in transcription regulation in humans (list from AMIGO version: 2.1.4 [18])

**Fig. 2:**
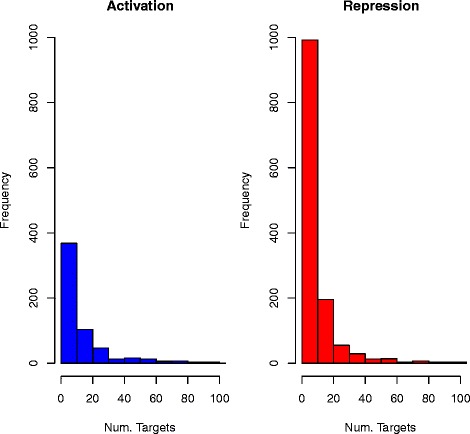
Histograms of the frequency of the number of predicted targets for the regulators in the database, for activation and repression (x-axes truncated at 100 targets, the maximum number of targets for activation is 245 and for repression 206)

Figure 2 shows a histogram of the frequency of the number of predicted targets, for activation and repression (x-axes truncated at 100). The figure indicates that there is a noticeable difference between the number of predicted targets for activation and for repression, which is discussed in a later section.

#### Target clusters

**Fig. 3:**
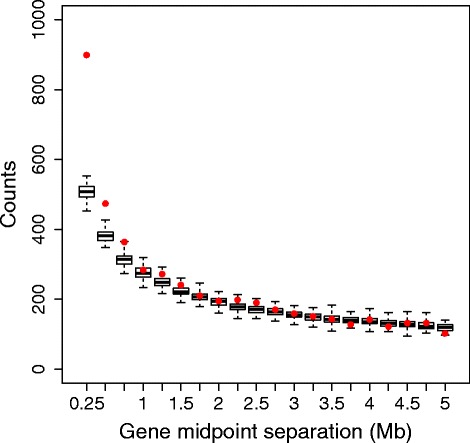
Plot showing the number of counts in 0.25 Mb bins when the genomic separation of target genes are analysed. The boxplot shows the results for random selections of genes (whiskers mark extreme values) and the dots mark the values obtained from the actual data

There is evidence that some of the predicted targets for some regulators form spatial clusters, that is, they are found in close proximity on a chromosome. For each regulator in the data set, if the regulator had more than one predicted target located on a particular chromosome, we took the mid point of each target and recorded the distance between adjacent targets. We repeated the analysis but replacing the predicted targets by the same number of genes selected at random from the same chromosome, and we repeated the randomisation analysis 100 times. Figure 3 shows the results. All the recorded distances have been pooled and divided into 0.25 Mb bins and the number of entries in each bin counted. The figure shows a boxplot of the counts in each bin from the 100 sets of randomised data, the boxplot whiskers marking the extreme values. Superimposed on the graph are the counts for each bin from the actual data. The figure shows that there are more predicted targets located closer together than you would expect to find at random, suggesting that some of the targets are forming locational clusters.

#### Chromosome

**Fig. 4:**
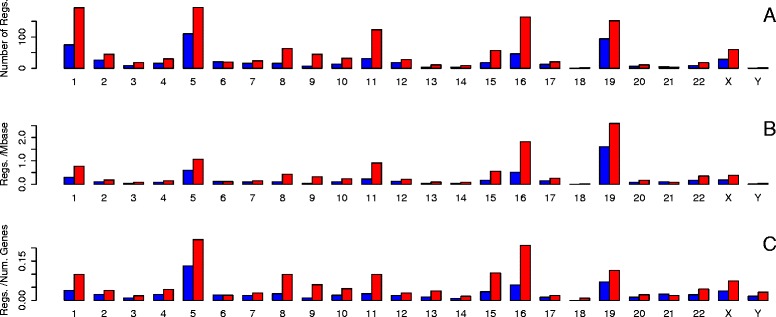
Number of regulators in the database located on each chromosome (blue = those with activation lists, red = those with repression lists) **a** Number of regulators **b** Density of regulators per Mbase **c** Number of regulators/Number of known genes on chromosome

**Fig. 5:**
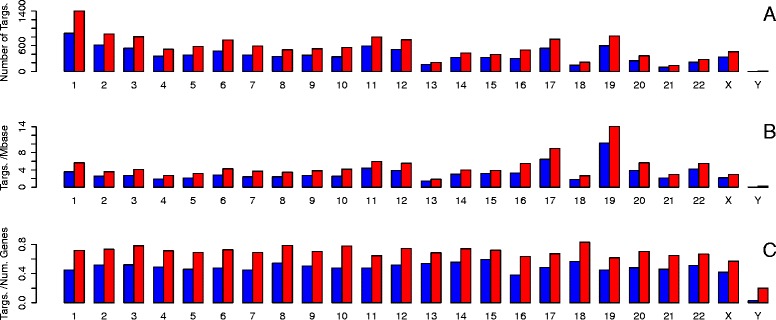
Number of predicted targets in the database located on each chromosome (blue = those activated, red = those repressed) **a** Number of targets **b** Density of targets per Mbase **c** Number of targets/Number of known genes on chromosome

Figure 4a shows a bar chart of the number of regulators on each chromosome (separated into activation and repression). 60 % of all regulators in the database lie on chromosomes 1, 5, 11, 16 or 19. When the numbers are corrected for chromosome length, chromosome 19 has the highest density of regulators in the database (Fig. 4b). When the numbers are corrected for the number of known genes on each chromsome, chromosomes 5 and 16 dominate (Fig. 4c). Variation of the density of regulators between chromsomes recorded in the database could be due to two different underlying causes. Firstly it could reflect an actual variation in the density of regulators between chromosomes. Secondly it could arise from variation in genomic instability between chromosomes. The nature of the analysis means that higher genomic instability will in general reveal more potential regulators. Figure 5a shows a bar chart of the number of targets per chromosome. There is a considerable variation in the number of targets on different chromosomes with a maximum of 2288, a minimum of 15 and a mean of 927. However when the numbers are corrected for the number of known genes on each chromosome the density of targets shows far less variation between chromosomes (Fig. 5c).

### Cocitations

Of the 1430 regulators in the database 10 % have cocitations with at least one of their predicted targets. Of the 22,255 predicted regulator-target pairs in the database 1 % have at least one cocitation; 35 cocitations being the maximum for any regulator-target pair. The low percentage of regulator-target pairs that have co-citations probably reflects the current relative paucity of evidence for the function of genes, with only a small percentage of genes having direct experimental evidence for their function ([19]).

As well as looking for regulator-target cocitations we looked at cocitations between the genes in a list of targets predicted for a regulator. For example POGZ has cocitations with two of its predicted targets (SP1 & XRCC6), but in addition, many of the 218 predicted targets are cocited together in papers. The maximum number of targets cocited in one paper is 6 but there are many different combinations of targets cocited in a total of 411 papers so altogether 65 % of the 218 targets are cocited with at least one other target. We ran a resampling to see whether this proportion of cocitations in a random list of genes of the same length could occur by chance and found it had a *p*-value of 0.0002. For TERF2IP, of the 380 predicted targets 80 % are cocited with at least one other target in at least one paper, with a *p*-value of 0.00001.

**Fig. 6:**
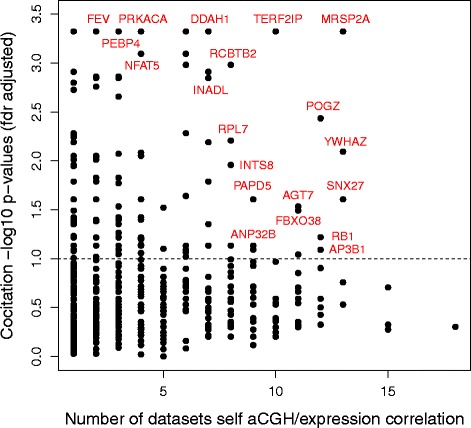
Scatter plot of target list cocitation adjusted *p*-values (-log10) for all the 1430 regulators in the database against the number of data sets in which the regulator shows significant self aCGH/expression correlation. The plot has been annotated with some of the regulators with the most significant cocited target lists and the largest number of data sets

Analysing the target lists of all regulators, 523 (37 %) of the 1430 regulators had cocitations of some of their target list genes and 135 of these were significant (adjusted *p*-value < 0.1). Figure 6 shows a scatter plot of target list cocitation adjusted *p*-values (-log10) for all the 1430 regulators against the number of data sets in which the regulator shows significant self aCGH/expression correlation (the target lists for activation and repression have been combined for any given regulator). The plot has been annotated with some of the regulators with the most significant cocited target lists and the largest number of data sets. The database gives a network graph for each regulator showing how cocitations in the literature, where they exist, link together the genes in the target list (activation and repression combined). The figure in Additional file 3 shows an example for the regulator POGZ.

### Activation/repression

There is a marked difference in the number of regulators repressing targets compared with the number activating targets (see Fig. 2). Altogether 104 regulators only activate targets but 841 regulators only repress targets; 485 regulators both repress and activate targets. There are in total 9088 regulator-target pairs showing activation and 13,167 regulator-target pairs showing repression in the database. An extra 45 % on the number of regulator-target pairs demonstrating activation are demonstrating repression. This suggests that inter-chromosomal acting regulatory relationships causing repression are more common than those causing activation. Intuitively this would seem plausible given the importance of feedback control in maintaining the stability of complex systems.

There is some evidence in the summary statistics from the database which suggests the effect is a true effect. Dividing the regulators into three groups, those which are predicted to activate and repress (485), those that are predicted to only activate (104) and those that are predicted only to repress (841) and examining the cocitations of the genes in the regulators’ target lists. For the 485 regulators which are predicted to activate and repress, 224 have cocitations of their activation target list and 212 of their repression target list. If a large number of the repression relationships were artefact then we would not expect similar numbers of lists to cocite. For the 104 regulators predicted only to activate 17 (16 %) have cocitation of target lists compared to 182 (22 %) of the 841 regulators predicted only to repress. Again if many of the repression relationships were artefact then we would not expect similar percentages to have cocitations of target lists. Other aspects of the results also show a consistency between activation and repression, for example, analysing the data for target clusters, as described in a previous section, but analysing activation and repression targets separately, gives very similar results (not shown). Also examining the number of regulators in the activation and repression lists which are known to be transcription factors (TFs), 12 % of the 104 regulators which purely activate are TFs and 15.0 % of the 841 regulators which purely repress are TFs.

## Discussion and conclusions

We have predicted inter-chromosomal regulator-target relationships from 31 publicly available matched aCGH/expression data sets for 1430 potential regulators. 206 of these are known to be involved in transcription regulation, although our definition of regulatory relationship extends beyond the direct relationship of transcription factor on a target to encompass very indirect relationships, through a pathway containing many intermediate regulatory steps. There is evidence that some of the targets of regulators are clustered by genomic location. The cocitations found for many of the target lists lends support to the predictions contained in the database.

The most striking observation from the results is the difference in the number of relationships involving activation and repression, and we present statistics from the database that suggests this bias is a true effect. It seems plausible that a complex dynamic system would require a preponderance of repression over activation in order to provide feedback control and maintain stability. There are however a number of other possible reasons for the observation. One potential cause of course is that it is an artefact of the analysis, however using Spearman correlation, with separate positive and negative null distributions, should avoid the introduction of any bias. Another hypothesis would be that amplification of some genes may be causing a sufficient elevation in the concentration of their transcript to result in excessive non-specific binding to the genome, disrupting and reducing the expression of genes not normally regulated by these amplified genes. We could also postulate that amplification of the DNA of a gene may result in elevated expression of the gene’s mRNA, but the sequence of the transcript is in some way deviant resulting in incorrect translation. In this way genes, which appear to be amplified, with correlated increases in expression, would actually have reduced levels of their proteins to activate targets, thus giving the impression of repression. Furthermore the difference in the numbers of activation and repression relationships could reflect differences in how the cell responds to the genomic disruption commonly found in cancer, rather than representing actual differences in levels of activation and repression in a non-disrupted cell. Finally, due to noise and tissue heterogeneity, the meta-analysis is only picking out a fraction of all regulatory relationships, so there is the possibility that the activation/repression bias may only occur in these relatively strong and more ubiquitous relationships selected by the analysis.

The results are available in the METAMATCHED database. We anticipate that the predictions contained in the database should be useful in constructing networks of regulatory relationships, informing experiments and perhaps in helping to predict downstream effects of drugs on their targets.

It should be noted that although cancer data sets are being used, this analysis is unlikely to pick out oncogenes which are consistently amplified or deleted in cancer samples. If a potential regulator is amplified or deleted in all the samples in a data set, and by the same amount, it probably will not have a high self aCGH/expression correlation. The genes with the highest self aCGH/expression correlation will be those that show a wide variation of copy number between samples in any given data set, with concomitant changes in expression. It is interesting to note that there are genes which do show this wide variation of copy number between samples in *multiple* data sets. One gene, MED4, has significant self aCGH/expression correlation in 18 datasets, and there are 120 regulators with significant self aCGH/expression correlation in 10 or more data sets. Perhaps these genes are located in genomic regions which are prone to disruption in cancer cells, but this disruption occurs erratically. Another reason might be that they occur in genomic regions which are consistently disrupted, but in later stages of the cancer development, and the data sets contain samples from a range of stages. If this is the case then some of these regulators may have an oncogenic role in later phases of the disease.

Whereas self aCGH/expression correlation can be consistent over many of the data sets, regulator-target correlations are significant in fewer data sets. This is partly due to noise in the experiments, but also suggests the relationships are rather specific to tissue type and pathology, and can be obscured by biological phenomena such as pathway remodelling and epigenomic effects. It is interesting to note that the regulator-target relationships identified in this study are likely to be gene regulatory relationships which are particularly susceptible to copy number disruption. They are not relationships which are protected from such disruption by alternative pathways and other buffering mechanisms. This may be important if any of these regulators do have an oncogenic role in later stages of cancer development.

Consistent coamplification or codeletion of neighbouring regulators and noise in the data can lead to ambiguity in the results as to which of the regulators is regulating a particular target gene. However in the database we do provide information beyond the best predictions, indicating what alternatives are suggested by the data. The results are of course constrained by the probes available on the arrays used in the matched experiments, further relationships are likely, but hidden by the absence of appropriate probes on the arrays.

It is not impossible that some of the predicted relationships in this study have arisen through a confounding factor as part of one of the many little understood or as yet unknown genetic mechanisms. For example there is now evidence that histone modification can promote copy number variation [20, 21]. If a histone modification was causing copy number variation in a regulator gene and the root cause of this histone modification was also affecting the expression of a second gene on a separate chromosome then this analysis might identify the second gene as an inter-chromosomal target of the regulator (provided the copy number of the second gene was not also affected, since we filter out these instances). Similarly there is some evidence from protozoa that copy number can be affected by RNA-mediated epigenetic effects [22], which suggests another potential route for confounding if this was found to occur in the human genome. So as well as detecting inter-chromosomal relationships arising from direct interactions, or indirect ones through pathways, the analysis may be picking up far more complex and subtle genomic effects, which cannot be isolated with our current state of knowledge.

There are a number of possible avenues for further work that could be pursued with this data. We chose to structure the meta-analysis to highlight gene relationships which are found in the maximum number of data sets. Alternative approaches emphasising sensitivity over specificity are possible and these could be assessed using target list cocitations. Similarly using Pearson correlation might reveal relationships not evident using Spearman correlation. Having established that there is useful signal in the data using a relatively simple but robust statistical approach it would be interesting to explore more complex methods such as maximal information-based nonparametric exploration statistics [23], designed to cope with non-linearities in the data being analysed.

We plan to include further matched aCGH/expression experiments as and when they become publicly available. We will update the cocitations and GO annotations at each release of the R packages org.Hs.eg.db and GO.db, and it will be interesting to see how cocitation support for the predictions changes with time.

## Additional files

Additional file 1
**Spreadsheet summarizing the results for all 1430 regulators in the database with significant predicted targets.** (XLS 746 kb)

Additional file 2
**Detailed description of METAMATCHED database content.** (PDF 108 kb)

Additional file 3
**Network representation of the cocitations of the target genes predicted for one regulator, POGZ.** Any two genes in the network are connected if they are cocited together in at least one publication. (PDF 11 kb)
