# Supplementary material for: Investigating inter-chromosomal regulatory relationships through a comprehensive meta-analysis of matched copy number and transcriptomics data sets
Source: BMC Genomics. 2015 Nov 18;16:967. doi: 10.1186/s12864-015-2100-5 (PMC4650296; doi:10.1186/s12864-015-2100-5)
Supplement: Additional file 2 — Detailed description of METAMATCHED database content. (PDF 108 kb) [file 12864_2015_2100_MOESM2_ESM.pdf]

Detailed description of METAMATCHED database content

Richard Newton & Lorenz Wernisch

July 15, 2015

For further information please email: richard.newton@mrc-bsu.cam.ac.uk

The METAMATCHED database of inferred regulatory relationships is available at http://sysbio.mrc-bsu.cam.ac.uk/METAMATCHED. The database contains entries for 12674 potential regulatory genes. The entry for a gene can be found through a search box on the main page. 1430 of the potential regulators have significant predicted targets (significance level Benjamini-Hochberg (BH) adjusted p-value < 0.1), and these genes are listed as links on the main page. The website can also be used to access information in the database on 19391 genes as targets. When a particular gene is searched for from the main page links are provided to information on the gene as a regulator (if any) and information on the gene as a target.

Gene as a Regulator

Selecting information on a gene as a regulator gives summary statistics for the gene, namely:

1. The number of significant (fdr<0.1) target genes activated by the regulator (positive correlation of aCGH profile and expression).
2. The number of significant (fdr<0.1) targets that are repressed by the regulator (negative correlation).
3. The number of regulator-target pairs which have cocitations, and this number as a percentage of the total number of predicted targets.
4. The total number of papers which cocite at least two genes from the list of significant targets and the regulator, and the statistical significance of finding this number of cocites.
5. List of significant target genes activated by the regulator (each entry linking to the webpage giving information from the database on that gene as a target)
6. List of significant target genes repressed by the regulator (each entry linking to the webpage giving information from the database on that gene as a target)
7. Links to results files (see below)
8. Link to a zip file by which all the results files can be downloaded
9. List of papers cociting regulator-target pairs, each entry linking to the papers Pubmed page.
10. A network graph showing how cocitations (if they exist) link the genes in the target list (activation and repression combined). The regulator is also included in the graph if it has cocitations with any of the targets.

The results files

- `GENENAME_activation_sig.csv` - for activation of targets by the regulator, a spreadsheet that contains the significant predicted targets for the regulating gene (significance level Benjamini-Hochberg (BH) adjusted p-value < 0.1), for which the regulator is the 'best' regulator in the database by way of either minimum p-value and/or significant in most datasets. This spreadsheet will not be present if the algorithm has predicted no such significant targets. The column names of the spreadsheet are:

  `gene` = predicted target gene's HGNC symbol.

  `chr` = predicted gene's chromosome.

  `pos` = predicted gene's chromosome position.

  `pv` = p-value for the correlation between the regulator and target genes based on a meta-analysis of the datasets. The correlation is between the regulating gene's aCGH and the target gene's expression. (Note: only those datasets for which the regulator gene has significant self aCGH/expression correlation are included in the meta-analysis).

  `fdr` = as above but BH adjusted.

  `fdr.rev` = BH adjusted p-value for the correlation between the regulator and target genes based on a meta-analysis of the datasets. Unlike `fdr` this is the 'reverse' correlation i.e. the correlation is between the target gene's aCGH and regulator gene's expression. If the regulator gene's aCGH is significantly correlated with a target gene's expression (so significant `fdr`), but the target gene's aCGH is also significantly correlated with the regulator's expression (so significant `fdr.rev`), this would suggest that a predicted relationship between the two genes is spurious, either due to coincidentally similar aCGH profiles or some form of coamplification/codeletion. (Since only inter-chromosomal relationships are being considered the latter explanation is less likely.)

  `num` = number of datasets which show significant correlation (BH adjusted p-values < 0.05) between regulator gene's aCGH and target gene's expression.

  `best-pv` = The 'best' regulator in the database for this target based on the criterion of minimum p-value. If the regulator associated with this page is the best regulator then this entry will be blank.

  `best-num` = The 'best' regulator in the database for this target based on the criterion of significance in the most number of datasets. If the regulator associated with this page is the best regulator then this entry will be blank.

  `pubmed` = If the regulator associated with this page and the target are cocited then the pubmed id(s) for the paper(s) will appear here.

  These columns dealing with the results from a meta-analysis of the datasets are followed by columns giving the results for each individual dataset. The values are the BH adjusted p-values for the correlation between the regulator gene's aCGH and target gene's expression (Note: only those datasets for which the regulator gene has significant self aCGH/expression correlation are included in the analysis and shown on the spreadsheet).

- `GENENAME_activation.csv` - a spreadsheet that contains the significance of all possible targets in the database for the regulating gene; for activation of the target by the regulator (ranked by Benjamini-Hochberg (BH) adjusted p-values). So in this

spreadsheet no criterion for most probable regulator and no threshold on p-value have been applied. The column names of the spreadsheet are as above.

Then there are similar files to the above for repression of the target by the regulator (that is, negative correlation between the regulator gene's aCGH and target genes' expression), the files are called `GENENAME_repression_sig.csv`, `GENENAME_repression.csv`.

- `GENENAME_target_cocitations.csv` - a spreadsheet with cocitation information. If file `GENENAME_activation_sig.csv` is present then a cocitation analysis is performed on a gene list comprising the regulator gene and all of its predicted targets. All papers that co-cite at least two of the genes in the list are recorded. The column names are:

  `CITED GENES` = the genes from the gene list cocited in this paper.

  `Number of Genes` = the number of genes cocited in this paper.

  `PUBMED ID` = the Pubmed (http://www.ncbi.nlm.nih.gov/pubmed) ID of the paper. Full information about the paper can be found by entering the Pubmed Id in the 'search' box at Pubmed.

  `PAPER TITLE` = the paper title.

  `LINK to PAPER` = the URL of the paper.

- `GENENAME_GO.html` = Gene Ontology annotations of a gene list comprising the regulator gene and all of its predicted targets. This file will be blank if no significant GO annotations were found.

Gene as a Target

Selecting information on a gene as a target gives summary statistics for the gene, namely:

1. Predicted regulator, for activation of the target, based on the criterion of lowest p-value, followed in brackets by the fdr (Benjamini-Hochberg (BH) adjusted p-value).
2. Predicted regulator, for activation of the target, based on the criterion of significance in the most number of datasets, followed in brackets by the fdr (Benjamini-Hochberg (BH) adjusted p-value).

   NB. the criterion of lowest p-value in 1. above is the *unadjusted* p-value, not the fdr value. The fdr value for the predicted regulator based on the criterion of lowest p-value and the fdr value for the predicted regulator based on the criterion of significance in the most number of datasets can, in some cases, be the same.
3. Number of significant predicted regulators (activation) - due to coamplification/codeletion of genes in the genomic region of the actual regulator this may be a relatively long list.
4. Predicted regulator, for repression of the target, based on the criterion of lowest p-value, followed in brackets by the fdr (Benjamini-Hochberg (BH) adjusted p-value).
5. Predicted regulator, for repression of the target, based on the criterion of significance in the most number of datasets, followed in brackets by the fdr (Benjamini-Hochberg (BH) adjusted p-value).

6. Number of significant predicted regulators (repression) - due to coamplification/codeletion of genes in the genomic region of the actual regulator this may be a relatively long list.
7. The number of the significant predicted regulators cocited in at least one paper with this target.
8. A list of these papers if any.
9. Results files - see below.

The results files

- `GENENAME_activation_sig.csv` - for activation of the target by regulators, a spreadsheet that contains the significant predicted regulators (significance level Benjamini-Hochberg (BH) adjusted p-value < 0.1), for which the regulator is the 'best' regulator in the database by way of either minimum p-value and/or significant in most datasets. This spreadsheet will not be present if the algorithm has predicted no such significant targets. The column names of the spreadsheet are:

  `gene` = predicted regulator gene's HGNC symbol.

  `chr` = predicted gene's chromosome.

  `pos` = predicted gene's chromosome position.

  `pv` = p-value for the correlation between the regulator and target gene based on a meta-analysis of the datasets. The correlation is between the regulating gene's aCGH and the target gene's expression. (Note: only those datasets for which the regulator gene has significant self aCGH/expression correlation are included in the meta-analysis).

  `fdr` = as above but BH adjusted.

  `fdr.rev` = BH adjusted p-value for the correlation between the regulator and target genes based on a meta-analysis of the datasets. Unlike `fdr` this is the 'reverse' correlation i.e. the correlation is between the target gene's aCGH and regulator gene's expression. If the regulator gene's aCGH is significantly correlated with a target gene's expression (so significant `fdr`), but the target gene's aCGH is also significantly correlated with the regulator's expression (so significant `fdr.rev`), this would suggest that a predicted relationship between the two genes is spurious, either due to coincidentally similar aCGH profiles or some form of coamplification/codeletion. (Since only inter-chromosomal relationships are being considered the latter explanation is less likely.)

  `num` = number of datasets which show significant correlation (BH adjusted p-values < 0.05) between regulator gene's aCGH and target gene's expression.

  `pubmed` = If the target associated with this page and the regulator are cocited then the pubmed id(s) for the paper(s) will appear here.

  These columns dealing with the results from a meta-analysis of the datasets are followed by columns giving the results for each individual dataset. The values are the BH adjusted p-values for the correlation between the regulator gene's aCGH and target gene's expression (Note: only those datasets for which the regulator gene has significant self aCGH/expression correlation are included in the analysis and shown on the spreadsheet).

- `GENENAME_activation.csv` - a spreadsheet that contains the significance of all possible regulators in the database for the target gene; for activation of the target by the regulator (ranked by Benjamini-Hochberg (BH) adjusted p-values). The ranked list is thresholded at a p-value < 0.33 for practical purposes. If there are no entries with a p-value < 0.33 then the top 100 entries are given. The column names of the spreadsheet are as above.

  Then there are similar files to the above for repression of the target by the regulator (that is, negative correlation between the regulator gene's aCGH and target genes' expression), the files are called `GENENAME_repression_sig.csv`, `GENENAME_repression.csv`.

Interpreting the results files

Consistent coamplification or codeletion of neighbouring potential regulators coupled with the inherent noise in the data can lead to ambiguity in the database as to which of the regulators is actually regulating a particular target gene. So the results files contain columns giving the 'best' regulator in the database for each target based on the criterion of minimum p-value and the 'best' regulator in the database based on the criterion of significance in the most number of datasets. In addition each gene has a webpage containing information in the database on the gene *as a target*. This page contains spreadsheets giving all the significant regulators of the target gene in question.

NB. A note on cross-referencing. For any regulator and a predicted target, details of the relationship will be found on two spreadsheets. Firstly the regulator will have a spreadsheet of predicted targets in which the target will appear. Secondly the target will have a spreadsheet of predicted regulators in which the regulator will appear. The *unadjusted* p-value of the relationship between the regulator and target in the two spreadsheets will of course be the same. However the two fdr values (the Benjamini-Hochberg adjusted p-value) will in general be different since the multiple correction has been performed on two different lists of p-values.
