# Supplementary figures and images for: Investigating inter-chromosomal regulatory relationships through a comprehensive meta-analysis of matched copy number and transcriptomics data sets
Source: BMC Genomics. 2015 Nov 18;16:967. doi: 10.1186/s12864-015-2100-5 (PMC4650296; doi:10.1186/s12864-015-2100-5)

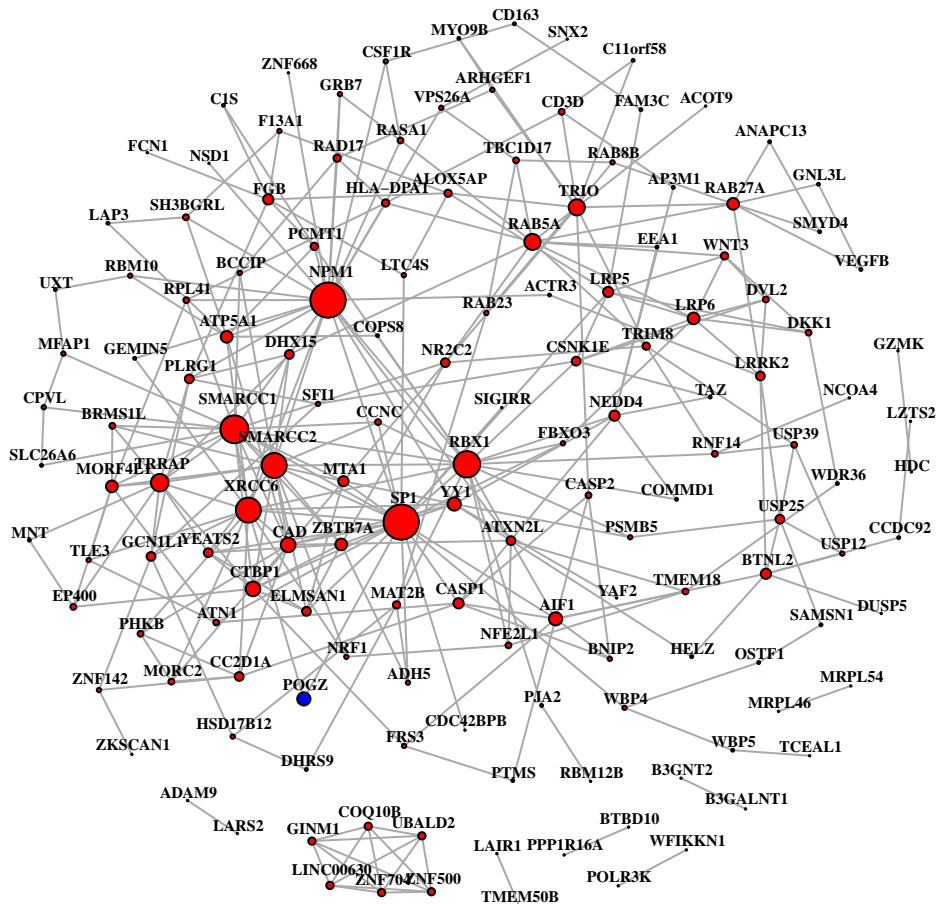

Supplement: Additional file 3 — Network representation of the cocitations of the target genes predicted for one regulator, POGZ. Any two genes in the network are connected if they are cocited together in at least one publication. (PDF 11 kb) [file 12864_2015_2100_MOESM3_ESM.pdf]
